# Using a Fiber Loop and Fiber Bragg Grating as a Fiber Optic Sensor to Simultaneously Measure Temperature and Displacement

**DOI:** 10.3390/s130506542

**Published:** 2013-05-16

**Authors:** Yao-Tang Chang, Chih-Ta Yen, Yue-Shiun Wu, Hsu-Chih Cheng

**Affiliations:** 1 Department of Information Technology, Kao Yuan University, Kaohsiung City 821, Taiwan; E-Mail: t10066@cc.kyu.edu.tw; 2 Department of Electrical Engineering, National Formosa University, Yunlin 632, Taiwan; E-Mail: ctyen@nfu.edu.tw; 3 Department of Electro-Optical Engineering, National Formosa University, Yunlin 632, Taiwan; E-Mail: chenghc@nfu.edu.tw

**Keywords:** fiber Bragg grating, fiber loop, temperature, displacement

## Abstract

This study integrated a fiber loop manufactured by using commercial fiber (SMF-28, Corning) and a fiber Bragg grating (FBG) to form a fiber optic sensor that could simultaneously measure displacement and temperature. The fiber loop was placed in a thermoelectric cooling module with FBG affixed to the module, and, consequently, the center wavelength displacement of FBG was limited by only the effects of temperature change. Displacement and temperature were determined by measuring changes in the transmission of optical power and shifts in Bragg wavelength. This study provides a simple and economical method to measure displacement and temperature simultaneously.

## 1. Introduction

Recently, with the rapid development of optical communications, numerous active and passive optical components have been developed, including wavelength division multiplexers (WDMs) and fiber Bragg gratings (FBGs). Fiber-optic sensors possess several advantages, including small size, light weight, high bandwidth, the ability to measure multiple points, the ability to withstand electromagnetic interference, and high operating temperature range. Fiber optic sensors have been used for various applications, such as medical endoscopes, chemical solution concentrations, and the measurement of the area of important components inside the mechanical precision measurement system.

A fiber optic sensor employs light to carry messages; this light passes through an optical fiber to a sensing area. The resulting changes in physical quantities in the sensing area (e.g., changes in strain, temperature, or refractive indices) can cause optical signals to transform, including the wavelength, power, and phases of these signals. Transformations in optical signals, in turn, can be employed to measure physical quantities. FBG is often used in fiber optic sensors and is developed by shining UV laser light on the fiber core with sub-micron modulations of the refractive index. This establishment of FBG can be achieved using various technologies, among which the phase masking technique is the most popular. Because of the invention of Bragg grating, fiber optic sensors can be developed for more numerous applications. For example, sensor heads can be used to measure a wide variety of physical quantities (e.g., strain, temperature, vibration, pressure, acceleration, and voltage) and can be applied for sensor multiplexing and sensor signal processing. In addition, multi-parameter fiber-sensing techniques are attractive for various reasons, including low loss, high bandwidth, immunity to electromagnetic interference (EMI), small size, light weight, safety, comparatively lower cost, and low maintenance requirements [1–7].

Although FBGs possess numerous advantages and wide applications, FBG measurements encounter a problem in that reflection wavelength displacement FBG is caused in the because of changes in strain and temperature. Thus, when the FBG is used as a sensor head, frequent and simultaneous changes in temperature and strain can lead to the integration of temperature and strain in the FBG, causing the two factors to become indistinguishable to the grating. The easiest way to overcome this problem and separately measure temperature is to employ special packaging around the sensing element; in this way, the inside of FBG does not cause wavelength drift because of changes in external strains. Patrick *et al.* [2] placed an FBG in the hollow of a steel casing or silica tube, with both sides of the FBG affixed to the casing using high-temperature resistant epoxy glue; as a result, FBG was isolated from strain. However, if both temperature and pressure were to be determined simultaneously, two FBGs with special settings or other auxiliary components were required. Lo [3] attached a single FBG to a cantilever by first pulling on both ends of the grating before releasing them after the FBG had been affixed to the cantilever. Reflection wavelength from non-adhered FBG was used to measure temperature change, and adhered grating was used to measure strain as well as temperature change. Temperature change measured using non-adhered grating was subtracted from the center wavelength displacement measured using the adhered grating to achieve a simultaneous measurement of temperature and strain. Jung *et al.* [4] proposed that FBG could be used with an erbium-doped fiber amplifier (EDFA) to simultaneously measure temperature and strain. This system employed declines in linear changes of spontaneous radiated power from an EDFA and increases in temperature to verify temperature measurements. Strain was verified by subtracting the temperature-affected grating wavelength displacement. In the study by [5], a special distributed-feedback fiber laser with four modes was used to measure strain and temperature simultaneously, which is smart and compact. However, this system had several drawbacks; for example, a fiber laser with four modes is difficult to fabricate, and an expensive high-frequency photodetector (PD) and electrical spectrum analyzer (ESA) are required. In a later study, the authors extended the basic concept to the simultaneous measurements of strain and temperature distributions using four FBGs [6]. Although the methods proposed in [6] proved successful, the computational time of the genetic algorithm was lengthy because examining the reflection intensity spectra of two or more FBGs was necessary to obtain the arbitrary strain and temperature profiles. Lai *et al.* proposed obtaining simultaneous measurements of the level and specific gravity of a liquid by using a dual-optical-fiber-sensor system comprising a fiber Bragg grating (FBG) level sensor and a Fabry-Pérot (FP) pressure sensor [7]. The experimental results showed that the average measurement errors of the dual-sensor system for the liquid level and specific gravity were 0.0323 and 0.0528 m, respectively.

Polymer FBGs have recently been widely used in fiber strain-sensor systems because of their low Young modulus and the high elasticity limit of the polymer fibers [8]. A simple fiber-strain sensor was presented using two cascaded polymer FBGs to cancel the thermal effect in the experiment; it successfully obtained the temperature-free strain recovery results and demonstrated the high wavelength tune range (12 nm with 2.25% strain) of the polymer FBGs [8]. The wavelength-based detection method offers various advantages, including simplicity, more economical polymer FBGs, and robustness in the optical-source power variation. In [9], the authors compared the polymer FBG accelerometer and silica-based FBG accelerometer. They showed that the polymer FBG accelerometer has superior sensitivity to the silica-based FBG. A temperature-compensated strain sensor can also be devised based on the wavelength detection methods by using two cascaded Sagnac interferometers and solid birefringent hybrid photonic crystal fibers (PCFs) [10]. The experimental results showed the high-strain and low-temperature sensitivities. However, the optical path difference of the two Sagnac interferometers must be well aligned, and using PCFs is more costly compared to previous methods employing FBGs.

In this study, a fiber loop, manufactured by using commercial fiber (SMF-28, Corning), and FBG were combined to form a fiber-optic sensor capable of the simultaneous measurement of displacement and temperature. The principle of optical fiber bending loss and temperature-affected FBG wavelength, which possess excellent linear change characteristics (as shown in the spectrogram), were used to measure changes in optical power loss and wavelength displacement and produce a simultaneous measurement of displacement and temperature. Compared to [8], the proposed method used a cascaded fiber loop and the FBG to achieve the simultaneous measurement of displacement and temperature; this is more economical and simpler because a fiber loop (manufactured using commercial fiber) is used. Moreover, the architecture of the proposed method is less complex for the fiber-strain sensor of the cascaded Sagnac interferometers [11].

## 2. Sensing Principles and Architecture

In this study, we employed a combination of FBG and a fiber loop to achieve a simultaneous measurement of temperature and displacement. The grating period of FBG was changed using temperature and strain, which caused displacement of the reflected Bragg wavelength. The equation demonstrating this relationship is as follows:

(1) $$\Delta \lambda_{B}=2n\Delta \Lambda$$

where Δλ_B_ is the change in Bragg wavelength, *n* is the effective refractive index, and ΔΛ is the change in grating period. Because this study employed FBG to measure temperature, Bragg wavelength was affected by the thermal expansion coefficient of grating and the thermal breakdown of optical fiber [3]. The equation demonstrating this relationship is as follows:

(2) $$\lambda_{B}=\lambda_{B0}+\lambda_{B0}\;\left(\xi +\alpha_{F}\right)\Delta T$$

where ξ is the thermo-optic coefficient of fiber material, α_F_ is the thermal expansion coefficient of FBG, and ΔT is changing temperature.

**Figure 1.**
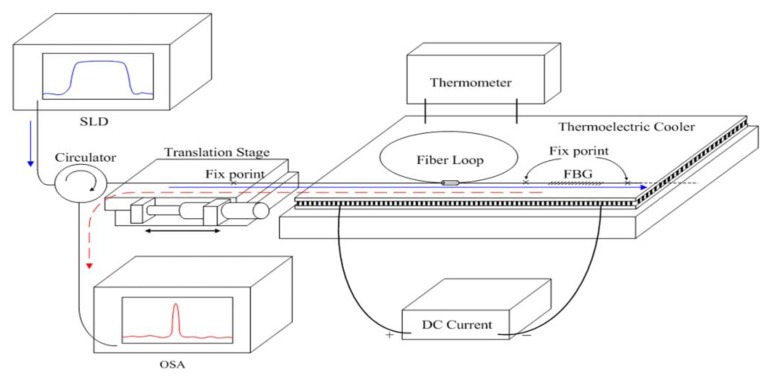
Experimental architecture diagram.

To test the practicality of our sensors, we affixed the FBG to a thermoelectric cooling module to isolate the effect of FBG displacement, as the architecture diagram in Figure 1 shows. Because experimental settings were employed, factors affecting the temperature sensitivity coefficient for FBG varied. If FBG was not affixed or tied to a particular object, factors affecting the temperature sensitivity coefficient included the thermo-optic coefficient (ζ) and the fiber thermal expansion coefficient (α_F_). Because, during the experiment, both ends of the FBG were affixed to the thermoelectric cooling module, the thermal expansion coefficient (α_S_) for the thermoelectric cooling module was also a factor affecting the temperature sensitivity coefficient for FBG. Therefore, the relational equation for temperature and FBG wavelength can be obtained by modifying Equation (2) to become the following equation:

(3) $$\lambda_{B}=\lambda_{B0}+\lambda_{B0}\;(\xi +\alpha_{F}+\alpha_{S})\Delta T$$

**Figure 2.**
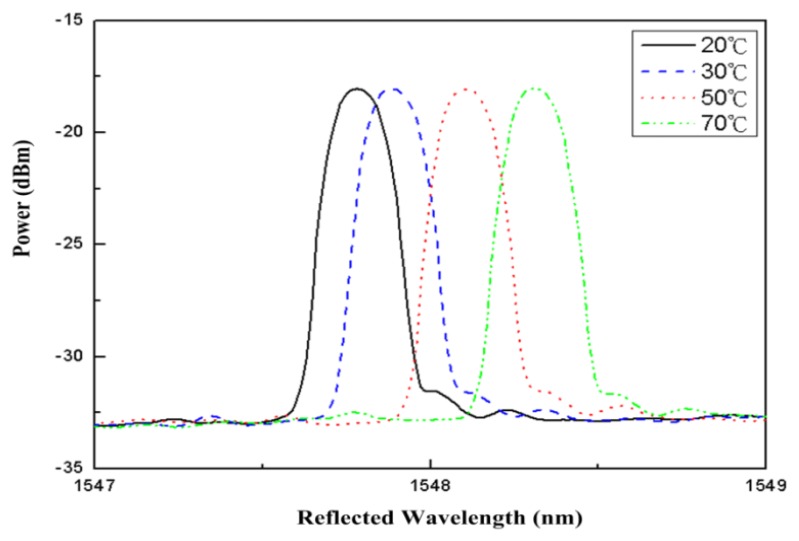
Center wavelength of the FBG at various temperatures.

Figure 2 shows center FBG wavelengths of 1,547.78 nm, 1,547.88 nm, 1,548.1 nm, and 1,548.31 nm (shown from left to right), which correspond to temperatures of 20 °C, 30 °C, 50 °C, and 70 °C, respectively. The fiber loop used in this study was composed of single-mode bare optical fiber with a circumference of 9 cm, which was wrapped in a 3 mm to 5 mm long plastic sleeve at the overlap area of the loop. Consequently, when either end of the fiber loop was subjected to tension, the loop maintained its shape, despite changes in size. The fiber loop was installed in a thermoelectric cooling module. One end of the loop was affixed to a mobile platform with a resolution of 10 μm, and one end was affixed to the thermoelectric cooling module. When the fiber in a loop is bent to a certain degree, the amount of signal lost increases in conjunction with increases in the degree to which a light signal is bent. This is the principle used when measuring displacement for a fiber loop. Assuming that light traveling through the bent section of the fiber loop causes optical power loss, ideal single-mode optical fiber output power is defined as follows [11]:

(4) $$P_{\text{out}}=P_{\text{in}}\exp(-2\alpha_{B}l^{e})$$

In this equation *l^e^*=*2πR^e^*, and *R^e^* is the effective bending radius that accounts for bending stress in the reflection coefficient of the fiber core material; therefore, *R^e^* is different from the actual diameter of the fiber loop. 2α_B_ represents the bending loss coefficient per unit length. When displacement is very small, the exponential function of displacement (Δd) in Equation (5) can be considered a linear function of Δd. Therefore, the relationship between optical power change and displacement can be rewritten as the following equation:

(5) $$\Delta P=-2\alpha_{B}P_{in}\Delta d$$

The results of Equations (4) and (5) show that the optical power change (ΔP) of the fiber loop is proportionally to the displacement (Δd).

Fiber must bend only to a certain degree before significant optical power loss occurs. However, this does not imply that further bending of fiber will lead to greater optical loss because light refracted in the fiber structure may be reflected back to the core layer, leading to increases in measured optical power. Therefore, linear optical power loss for a fiber loop must occur in a certain range.

**Figure 3.**
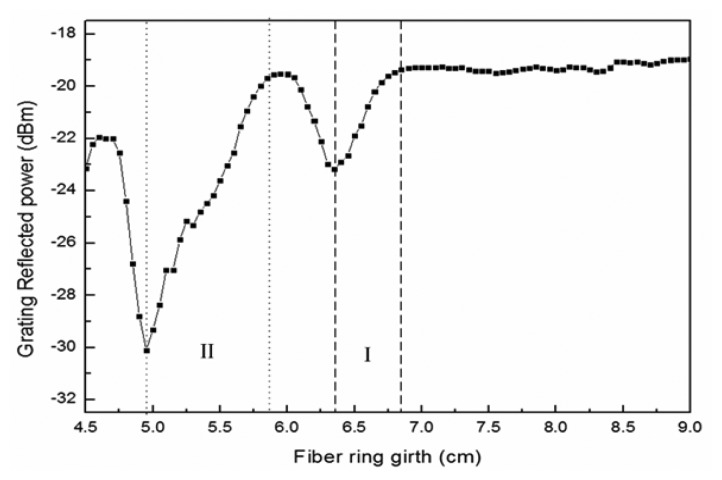
Changes in optical power for a fiber loop at various circumferences.

The fiber loop was manufactured using commercial fiber (SMF-28, Corning). Figure 3 shows the decrease, at room temperature, in the circumference of a fiber loop from 9 cm to 4.5 cm at a displacement increment of 0.5 mm as changes in optical power for the loop are measured and observed. The figure shows that fiber loop circumference in the ranges of approximately 6.35 cm–6.75 cm and 4.9 cm–5.8 cm coincide with locations in which power loss occurs in Figure 3. However, optical power loss in the second range is much greater because of temperature change, and, consequently, temperature and displacement are difficult to distinguish at this range. Thus, circumferences in the range of approximately 6.35 cm–6.75 cm were chosen as the application range for this study. Matrices were employed to represent the effect of grating wavelengths on changes in temperature and the effect of optical power on changes in displacement and temperature, as shown in the following equation:

(6) $$\left[\begin{array}{c} \lambda_{B}\\ P \end{array}\right]=\left[\begin{array}{cc} k_{T1} & k_{d1}\\ k_{T1} & k_{d2} \end{array}\right]\;\left[\begin{array}{c} \Delta T\\ \Delta d \end{array}\right]+\left[\begin{array}{c} \lambda_{0}\\ P_{0} \end{array}\right]$$

where λ_B_ and P represent the measured Bragg wavelength and optical power, respectively; k_T1_ and k_T2_ represent the temperature change sensitivity for FBG and the fiber loop, respectively; k_d1_ and k_d2_ represent the displacement change sensitivity for FBG and the fiber loop, respectively; and λ_B0_ and P_0_ represent the grating reflection wavelength and reflected power, respectively, before any change in temperature or displacement occurs. Because of the setup for the experiment, FBG was not affected by displacement; consequently, k_d1_ can be reduced to 0. Therefore, Equation (6) can be converted to the following:

(7) $$\left[\begin{array}{c} \lambda_{B}\\ P \end{array}\right]=\left[\begin{array}{cc} k_{T1} & 0\\ k_{T2} & k_{d2} \end{array}\right]\;\left[\begin{array}{c} \Delta T\\ \Delta d \end{array}\right]+\left[\begin{array}{c} \lambda_{B0}\\ P_{0} \end{array}\right]$$

If temperature and displacement sensitivity coefficients are known, Equation (7) can be employed to calculate amounts of temperature change and displacement as well as measure temperature and displacement. The relationship matrix for change in wavelength and power loss and temperature and displacement is expressed in Equation (8), where D=k_T1_k_d2_:

(8) $$\left[\begin{array}{c} \Delta T\\ \Delta d \end{array}\right]=\left[\begin{array}{cc} k_{d2} & 0\\ -k_{T2} & k_{T1} \end{array}\right]\;\left[\begin{array}{c} \Delta \lambda \\ \Delta P \end{array}\right]$$

## 3. Experimental Results

During the experimental measurements, a superluminescent diode (SLD) (ASLD155-200, Amonics) was used as the light source, and an Anritsu MS9710C was used as the spectrometer. The measured spectral range was 600 nm to 1,750 nm, and 0.1 nm was chosen as the resolution setting for the spectrometer. We first re-evaluated the temperature sensitivity coefficient for FBG, and ensured that both ends of the FBG were fixed to the thermoelectric cooling module and not affected by displacement effects. After these procedures, the temperature sensitivity coefficient for FBG was relatively easy to obtain. Power supply was used to provide currents to the thermoelectric cooling module, and current size was used to control temperature. The experiment employed 1,547.81 nm as the grating center wavelength and 0.21 nm as the bandwidth.

**Figure 4.**
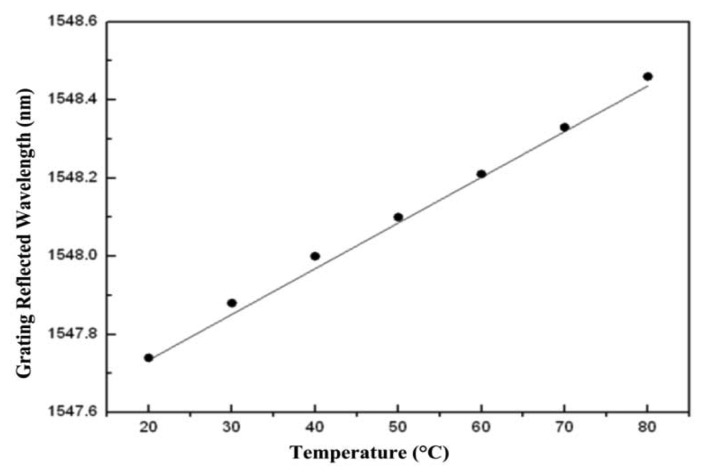
Changes in the center wavelength of FBG at differing temperatures.

**Figure 5.**
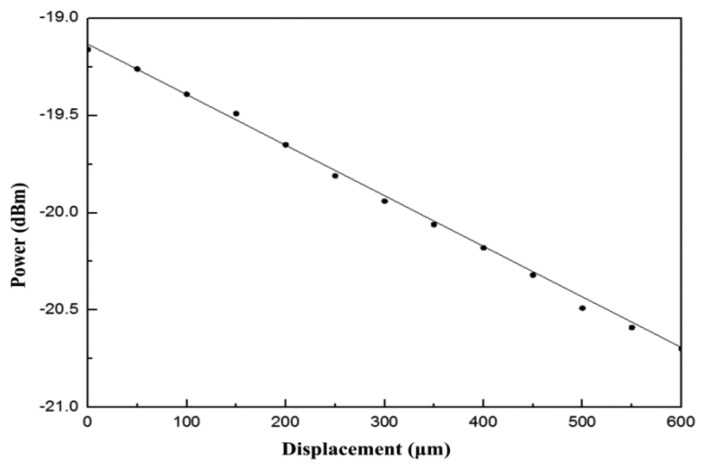
Changes in optical power as displacement increased at 50-μm increments at an FBG temperature of 70 °C.

**Figure 6.**
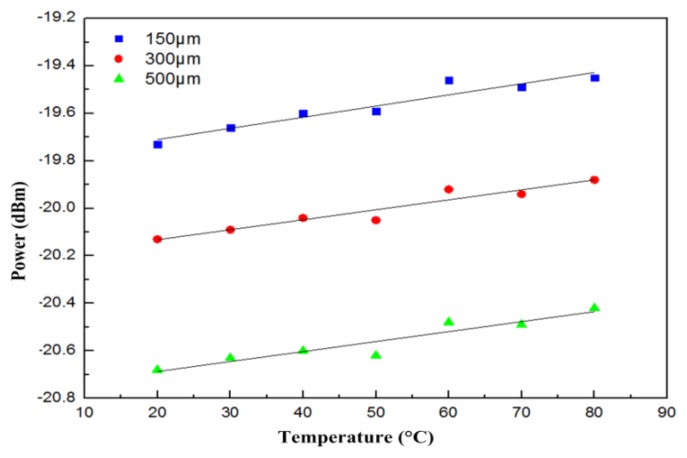
Changes in sensor optical power with displacement maintained at 150 μm, 300 μm, and 500 μm as different temperatures were applied.

The dots in Figure 4 represent various FBG wavelengths within a temperature range of approximately 20 °C to 80 °C. This 60-°C change in temperature resulted in a wavelength shift from 1,547.74 nm to 1,548.46 nm. The line portion represents the use of linear equations to simulate the curves closest to the wavelength displacements. Most wavelength displacements obtained 0.0117 nm/°C as the temperature sensitivity coefficient (k_T1_) for FBG. After temperature coefficients for FBG were obtained, temperature and displacement sensitivity coefficients for the fiber loop were determined. To obtain temperature sensitivity coefficients for different temperatures, displacement was rendered constant. By contrast, to obtain displacement sensitivity coefficients, temperature was rendered constant, with only displacement being allowed to change. Figure 5 shows optical power change at a displacement increment of 50 μm for various temperature environments for the fiber. The displacement sensitivity coefficient (k_d2_) for the fiber loop was −0.00274 dBm/μm. Figure 6 shows optical power change for the fiber loop at various temperatures, with displacement amounts maintained at 150 μm, 300 μm, and 500 μm. The obtained temperature sensitivity coefficient (k_T2_) was 0.0044 dBm/°C.

Displacement and temperature sensitivity coefficients were obtained for the fiber loop and FBG based on the experiment conducted in this study. We then used these coefficients to create the relationship matrix for optical power change and wavelength displacement and displacement and temperature change:

(9) $$\left[\begin{array}{c} \Delta T\\ \Delta d \end{array}\right]=\left[\begin{array}{cc} 0.0117 & 0\\ 0.0044 & -0.00274 \end{array}\right]\;\left[\begin{array}{c} \Delta \lambda \\ \Delta P \end{array}\right]$$

(10) $$\left[\begin{array}{c} \Delta T\\ \Delta d \end{array}\right]=\frac{1}{-3.21\times 10^{-5}}\left[\begin{array}{cc} -0.00274 & 0\\ -0.0044 & 0.0117 \end{array}\right]\;\left[\begin{array}{c} \Delta \lambda \\ \Delta P \end{array}\right]$$

**Figure 7.**
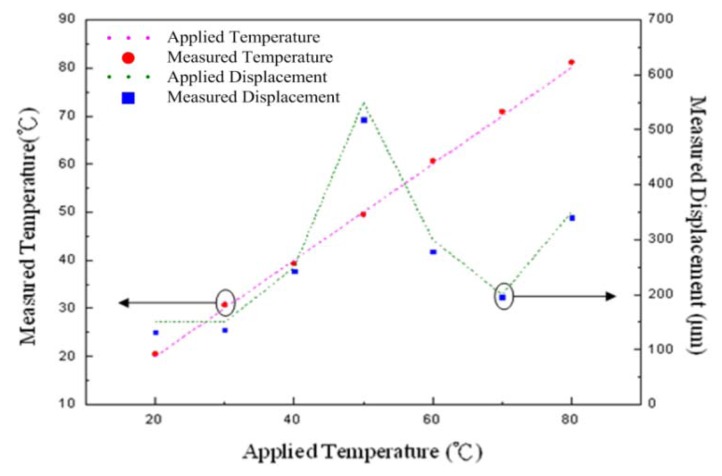
Comparison of actual displacement and temperature and measured displacement and temperature at various temperatures.

In the present study, we simultaneously measured temperature and displacement by first measuring the center wavelength displacement of FBG to determine changes in temperature. The effects of both temperature and displacement on the fiber loop caused it to lose optical power. However, displacement amounts can be measured by subtracting detected temperature change from center wavelength displacement. Therefore, the experiment simultaneously measured temperature and displacement through measurements of optical power and wavelength displacement. Figure 7 shows a comparison between the measured and applied values of temperature and displacement for the sensor at various temperature conditions.

When varying amounts of displacement for each temperature level were exerted, the root mean square errors for temperature and displacement for temperatures ranging from 20 °C to 80 °C were ±10.57 μm and ±0.78 °C, respectively. These results demonstrate that both temperature and displacement measurements were consistent with the actual temperatures and displacements applied to a sensor. Therefore, the experimental setup used to simultaneously measure temperature and displacement is feasible for real situations.

## 4. Conclusions

This study introduced the basic principles of FBG and the reasoning behind wavelength drift caused by the effects of temperature and strain on the center wavelength of FBG. The drifting of the center wavelength results from the influence of changes during the grating period, or from changes in temperature that lead to transformations during the grating period because of heating or cooling as well as changes in the effective refractive index at the core of FBG. Although change to FBG caused by temperature and strain is linear, other settings are required to distinguish the effect of temperature on FBG. Thus, in the present study, we combined a fiber loop and FBG in a sensor head to simultaneously measure temperature and displacement. We first measured changes in temperature using the displacement of the center wavelength of FBG. Factors that caused optical power loss included temperature and displacement. By simply subtracting the effect of temperature, which is measured using center wavelength displacement, from optical power loss, the sensitivity coefficient for optical power loss in the fiber loop and displacement could be obtained. Therefore, changes in temperature and displacement can be determined simultaneously by measuring center wavelength displacement and optical power loss. Finally, we verified that sensors demonstrate varying amounts of displacement at different temperatures. Results from comparing measured values against actual values showed that our proposed sensor setting is feasible for practical application to simultaneously measure temperature and displacement. The root mean square error for temperature and displacement was± 0.78 °C and ± 10.57 μm, respectively.
